# Comparison of 4 and 6 weeks of rest period for repair of root resorption

**DOI:** 10.1186/s40510-017-0173-1

**Published:** 2017-07-17

**Authors:** Sneh A. Mehta, Shailesh V. Deshmukh, Ravindranath B. Sable, Amol S. Patil

**Affiliations:** Department of Orthodontics and Dentofacial Orthopaedics, Bharati Vidyapeeth Dental College and Hospital, Bharati Vidyapeeth Deemed University, Dhankawadi, Pune, 411043 Maharashtra India

**Keywords:** Root resorption, Repair, Intrusion, Histology

## Abstract

**Background:**

The study was designed to evaluate and compare the rest periods of 4 and 6 weeks for healing of orthodontically induced root resorption craters.

**Methods:**

The study was conducted with a split-mouth design, with the right and left mandibular first premolars of 14 subjects serving as the two groups of the study. The right premolars constituted group A and the left ones, group B. Intrusive force was applied on these teeth for a period of 6 weeks, followed by retaining the teeth for 4 weeks (group A) and 6 weeks (group B) as rest periods before extraction. The extracted teeth were prepared for histologic examination with haematoxylin and eosin staining and studied under a light microscope. The histological sections were scored based on the level of repair (none, partial, functional, or anatomic) seen in the deepest craters in the apical third region of the roots. The mean values of the scores in the two groups were compared using Mann-Whitney *U* test.

**Results:**

All the teeth showed healing in their deepest craters. The teeth in group A showed partial repair more frequently (84.6%), with the remaining (15.4%) showing functional repair. The teeth in group B showed anatomic repair more frequently (60%), with the remaining (40%) showing functional repair. The mean level of repair was higher in group B (2.6 ± 0.5) as opposed to that in group A (1.15 ± 0.37). The difference between these values was of very high significance (*P* < 0.05).

**Conclusions:**

Longer rest period of 6 weeks showed more advanced healing than a shorter rest period of 4 weeks. Six weeks of rest period is adequate only for the functional repair of resorption craters.

## Background

Orthodontic tooth movement is always accompanied by some amount of root resorption [1, 2]. When the orthodontic force application is stopped, the active root resorption is known to transition into a process of repair. This repair is believed to be of three kinds: partial, functional, and anatomic [3]. Partial repair occurs when the exposed dentin is only partially covered by new cementum with some area of the exposed dentin remaining uncovered; functional repair occurs when the exposed dentin has been covered completely by a thin layer of repair cementum without the re-establishment of its original contour; and anatomic repair is characterized by the restoration of the root surface to its original contour.

Amount of root resorption has been studied extensively in relation to the magnitude as well as the duration of application of orthodontic forces [4, 5]. The general finding is that continuous forces are more detrimental as they lead to more amount of root resorption. Discontinuous forces (interrupted and intermittent) on the other hand allow for healing of these resorption cavities during the rest periods when the orthodontic force is not being applied [1, 2, 4, 5]. When resorption has reached appreciable levels, it is advisable to discontinue the orthodontic forces for some amount of time to allow for repair of the craters. However, few studies have quantified the amount of time for which the orthodontic force should be discontinued so as to permit full anatomic repair of the resorption craters. It would be beneficial to limit these rest periods to the shortest possible time so that it does not create a big impact on the total treatment time. Owman-Moll et al. suggest that only partial repair of the craters occurs by waiting for 4 weeks, while functional and anatomic repair predominate with a rest period of 5 to 8 weeks [3]. Cheng et al. observed that orthodontic resorption undergoes anatomic repair with a rest period of 8 weeks [6].

This study was an attempt to evaluate the following: (i) if a period of 6 weeks is enough for the orthodontically induced resorption craters to heal anatomically, (ii) the progress of repair from 4 weeks to 6 weeks after discontinuation of orthodontic forces, and (iii) the difference in the thickness of repair cementum after the two time periods as measured in micrometer.

## Methods

This study was conducted on the right and left mandibular first premolars of 15 subjects who reported for orthodontic treatment and who were indicated for extraction of mandibular first premolars for the same. Subjects were explained the procedure and informed consent was obtained from each participant. The mean age of the selected sample was 15.4 years ± 2.95 years (12–23 years). The study was approved by the ethics committee of the university.

In order to be included in the study, the subjects had to be without (i) any history of previous orthodontic treatment, (ii) trauma in the orofacial region, and (iii) predisposing systemic diseases. The mandibular first premolars had to be fairly upright as evident visually, free of any periodontal or endodontic pathology, and with completely closed apices and without pre-existing root resorption as seen on the orthopantomogram.

**Fig. 1:**
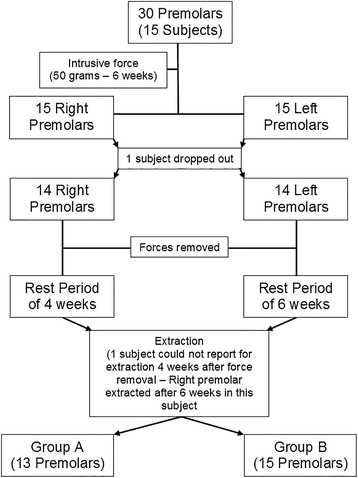
Design of the study

The study was a split-mouth study, with the right and left mandibular premolars of the subjects forming the two groups of the study, viz. group A, where the teeth were retained for 4 weeks post-orthodontic intrusion, and group B, where the teeth were retained for 6 weeks similarly. The final study design is shown in Fig. 1.

Mandibular first molars were banded with 0.018″ MBT tubes (3M Unitek, Monrovia, California). Occlusal grinding of the first premolars was done till enough clearance was obtained for bonding the spring on to the tooth. Interproximal clearance was obtained using single-sided abrasive strips to prevent damage to the adjacent tooth.

**Fig. 2:**
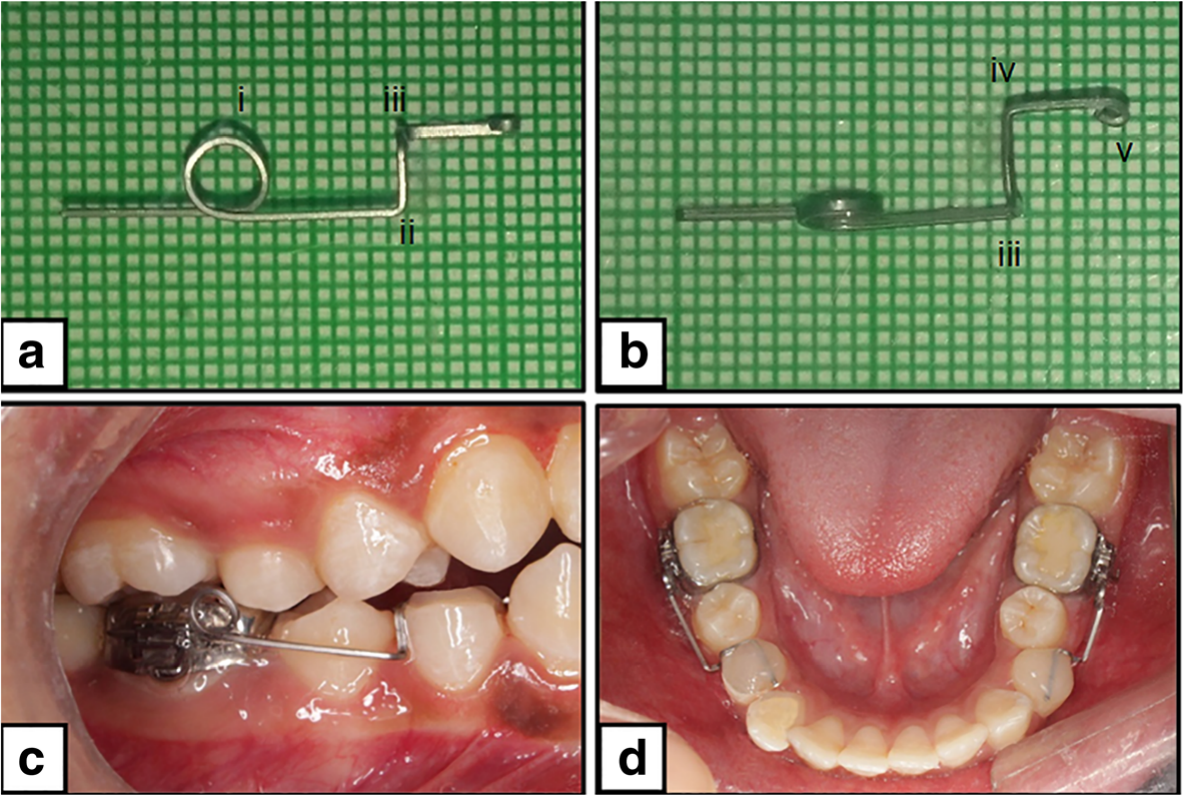
Constructed spring for a right mandibular first premolar. **a** Lateral view: (*i*) coil, (*ii*) occlusal bend, and (*iii*) lingual bend. **b** Occlusal view: (*iii*) lingual bend, (*iv*) anterior bend, and (*v*) end bent into a round. **c** Right lateral view of the bonded spring. **d** Occlusal view of the bonded springs

A spring design was chosen which involved a posterior coil and a cantilever arm resting on the occlusal surface of the mandibular first premolar to provide an apical direction of the force. Each spring was prepared using a 0.017″ × 0.025″ TMA wire (Ormco, Orange, California) in which a coil with 3-mm internal diameter was incorporated in front of the molar. The spring was activated before bonding to the premolar by opening the coil so that the active arm goes apically. The amount of activation was adjusted to a magnitude of 50 g of force as seen on a dial gauge. The spring was bonded onto the occlusal surface of the premolar (Fig. 2).

The active intrusion was carried out for a period of 6 weeks on both sides. These force levels and duration were used as they are physiologically feasible for premolar intrusion and have been shown to induce root resorption in human premolars [6–8]. The springs were then removed and deactivated so that no active force acts on the teeth once they are rebonded in the previous manner. At this point, the springs were only providing retention of the teeth. This was necessary to prevent further resorption with extrusive movements during relapse.

After completion of the allotted retention time (4 weeks for the right premolars and 6 weeks for the left premolars), the teeth were extracted atraumatically with forceps under local anaesthesia. The extracted teeth were immediately washed under running water and placed in formaldehyde solution diluted to a 1:10 ratio with distilled water and stored in this manner for a minimum of 5 days to allow fixation.

**Fig. 3:**
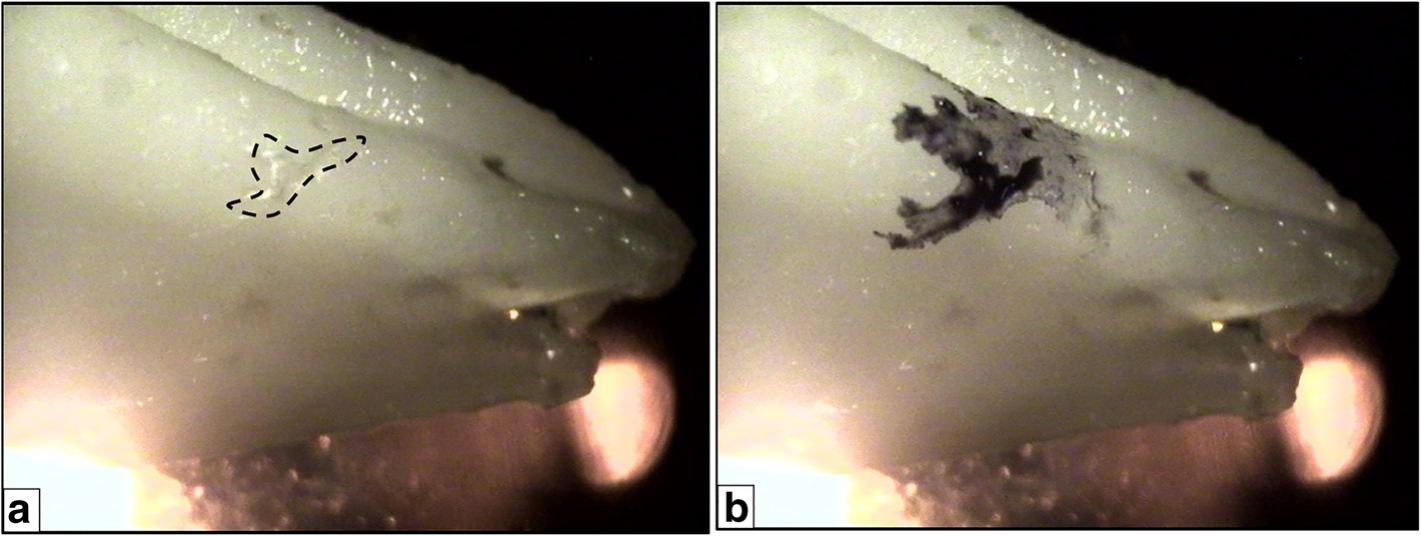
Irregularities seen on the root surfaces when observed under the stereomicroscope. The largest irregularity was selected [*dotted outline* in **a**] and marked **b** with India ink solution

The teeth were then prepared for histological sectioning. A stereomicroscope (XTL 3400E, Wuzhou New Found Instrument Co., Ltd, Guangxi, China) was used at a magnification of ×10 to study these teeth. After careful examination of each surface (buccal, lingual, mesial, and distal), the largest surface irregularity in the apical third of each tooth was marked using a toothpick dipped in India ink solution (Fig. 3). They were decalcified using 5% nitric acid solution for a period of 10 to 12 days. The specimens were then cut longitudinally into two halves with a scalpel through the areas marked previously with the India ink stain under the stereomicroscope, and one half was embedded in paraffin. Thin paraffin ribbons of a uniform thickness of 5 μm were obtained for further processing with a precision rotary microtome (HM 340E, Thermo scientific, Waltham, MA). These ribbons were then mounted on slides and stained with haematoxylin and eosin. The slides thus obtained were examined under a light microscope (DM 1000LED, Leica Microsystems Limited, Wetzlar, Germany). Histological photographs were obtained for examination using a digital microscope camera assembly (DFC 290HD, Leica Microsystems, Germany).

The repair cementum in each tooth was identified, delineated by reversal lines and differential staining. The deepest crater in the section, as verified by visual examination under the microscope, was selected for further analysis. In cases of conflict in deciding upon the deepest crater, an image analysis software (Leica Application Suite, v4.1.0, Leica Microsystems Limited, Heerbrugg, Switzerland), which could make linear measurements on histological images, was used to compare the depth of the craters from the bottommost point in the crater to the outer root outline imagined as a continuation of the adjoining cementum. The following parameters were studied for each of these craters undergoing repair.

### Level of repair

**Fig. 4:**
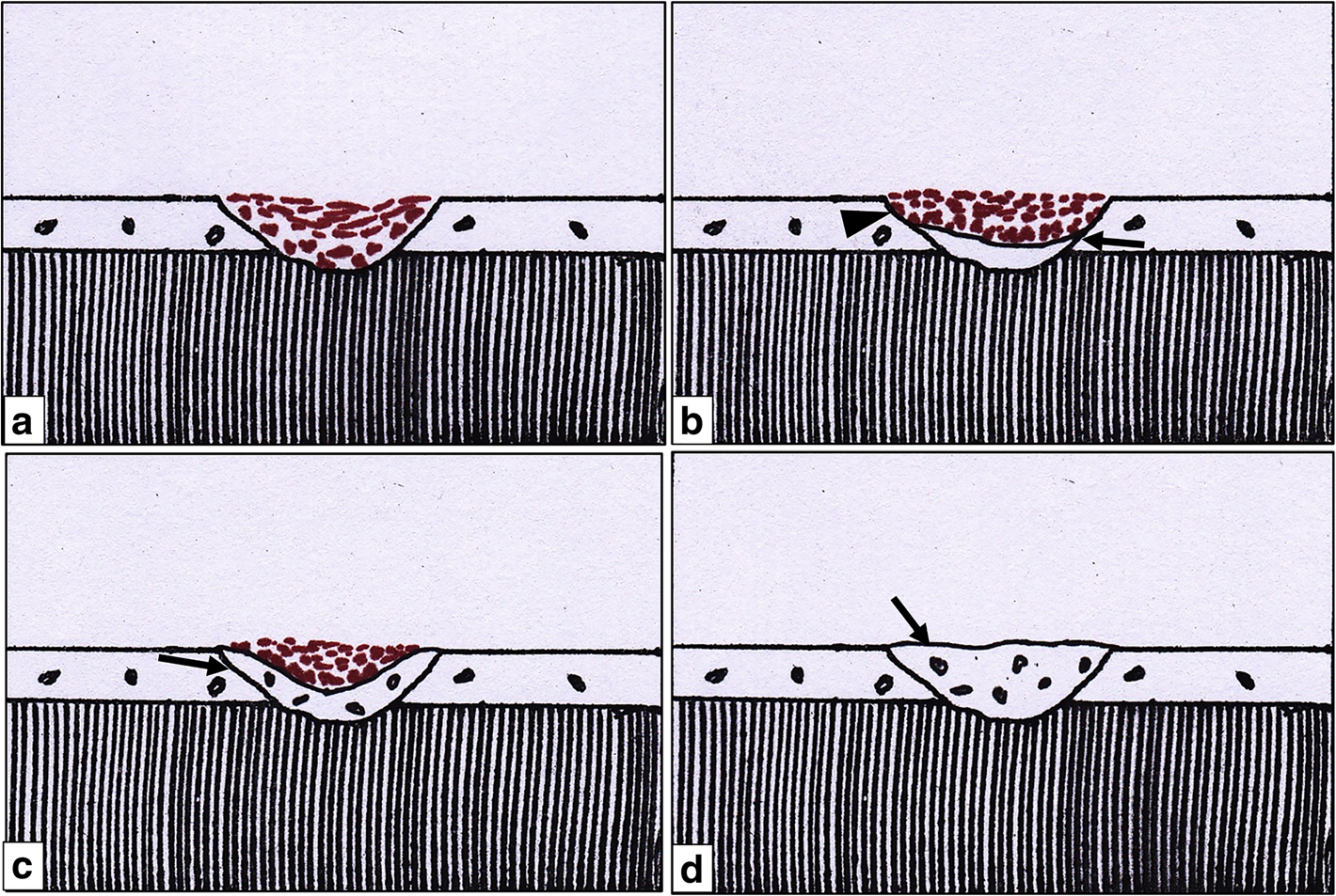
Schematic presentation of the level of repair cementum. Craters showing **a** no repair, **b** partial repair with only a part of the surface of the resorption cavity covered with reparative cementum (*arrow*) and the remaining surface exposed (*arrowhead*), **c** functional repair with the total surface of the resorption cavity covered with reparative cementum (*arrow*) without re-establishment of the original root contour, and **d** anatomic repair with the total surface of the resorption cavity covered with reparative cementum (*arrow*) to such an extent that the original root contour is re-established

The grading system described by Owman-Moll et al. [3] was used to quantify the level of repair of these craters (Fig. 4). Scores were assigned to each level of repair to facilitate the statistical comparison between the two groups. The scores were assigned as shown in Table 1.

**Table 1:** Scoring system for level of repair

Level of repair	Score
No repair	0
Partial repair	1
Functional repair	2
Anatomic repair	3

### Thickness of repair cementum

**Fig. 5:**
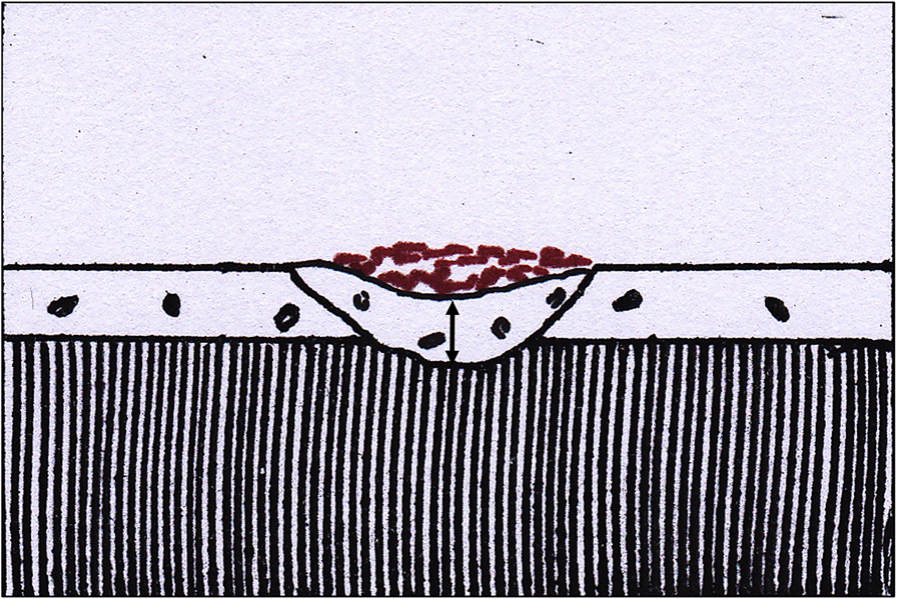
Schematic fig. to show the method of measuring repair cementum thickness. Measured as perpendicular distance from the deepest point of the crater till the outer surface of the layer (*double-headed arrow*)

The thickness of the repair cementum in these same craters was measured (in μm) as the perpendicular distance from the deepest point of the crater till the outer surface of the layer (Fig. 5). The image analysis software mentioned before was used for this purpose. Two measurements were taken by two different operators.

### Statistical analysis

Statistical tests were performed using R statistical software, v3.2.2. To check the reliability of the system utilized to measure the thickness of the repair cementum, the interoperator reliability for the cementum thickness readings was assessed using Spearman’s rank correlation coefficient for both the groups. Mean values and standard deviations were calculated for the scores of level of repair for both the groups and compared using Mann-Whitney *U* test. Similarly, mean values and standard deviations for the base readings (first operator) for cementum thickness obtained from both the groups were calculated and compared using Mann-Whitney *U* test. A “*P* value” of <0.05 was considered statistically significant for both the comparisons.

## Results

Fifteen subjects originally participated in the study. However, 1 subject dropped out midway before extraction of the mandibular first premolars. The final sample consisted of 14 subjects of which, 4 were males and 10 were females. Another subject could not report for extraction at 4 weeks after force withdrawal. Subsequently, both mandibular first premolars of this subject were included in group B, the 6 weeks rest period group. Subsequently, group A included 13 teeth and group B included 15 teeth. The largest craters that were analysed for the study were all found in the apical third of the lingual surfaces of the specimens.

**Fig. 6:**
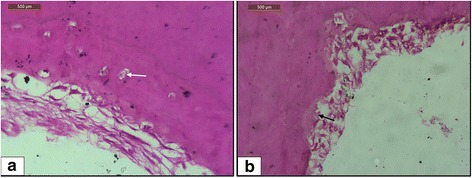
Light microscope view of different types of cementum in the repair tissue. Magnification of ×40. **a** Craters showing repair with cellular cementum. Cementocytes are seen embedded in lacunae (*white arrow*) within the cemental matrix. **b** Craters showing repair with acellular cementum (*black arrow*)

Histologically, all the teeth showed healing in their deepest craters. Healing was with either type (cellular/acellular) of cementum, with early repair areas showing the presence of acellular cementum predominantly (8 out of 13 in group A) and later areas showing the presence of cellular cementum more often (13 out of 15 in group B) (Fig. 6). The results of the study are presented below.

### Level of repair

**Fig. 7:**
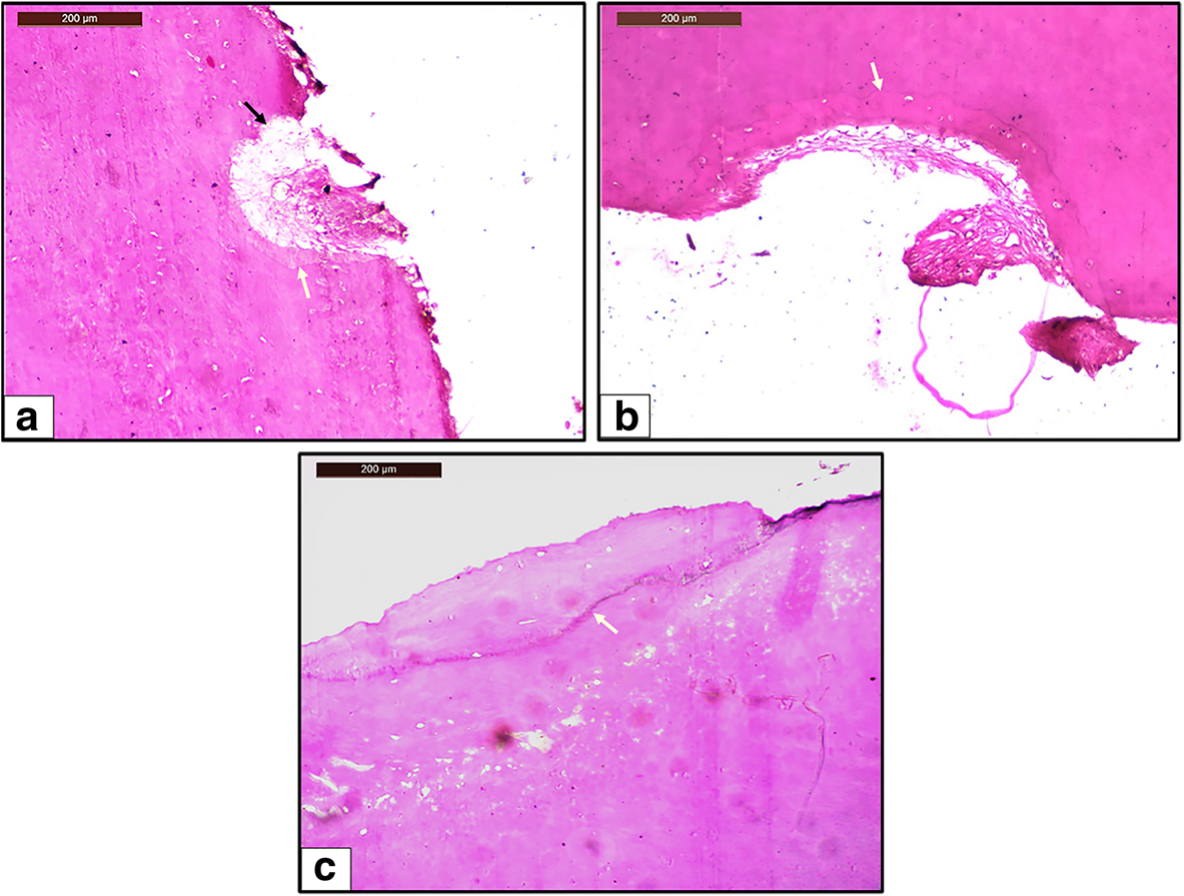
Light microscope view of different levels of repair. Magnification of ×10. **a** Partial repair of resorption crater seen in a tooth from group A. Only a part of the surface of the resorption crater is covered with reparative cementum, and the remaining surface is exposed (*black arrow*). **b** Functional repair of resorption crater seen in a tooth from group A. The entire surface of the resorption crater is covered with reparative cementum without re-establishment of the original root contour. **c** Anatomic repair of resorption crater seen in a tooth from group B. The entire surface of the resorption crater is covered with reparative cementum to such an extent that the original root contour is re-established. *White arrow* indicates the reversal line in each image

All the teeth showed healing in their deepest craters. Thus, none of the teeth in the sample were assigned a score of 0. A majority of the teeth in group A showed partial repair (score 1), with the remaining showing functional repair (score 2). A majority of the teeth in group B showed anatomic repair (score 3), with the remaining showing functional repair (see Fig. 7). The distribution of different levels of repair amongst the two groups is shown in Table 2. The same table shows that the mean level of repair in group A was 1.15 ± 0.37 and that in group B was 2.6 ± 0.5. Mann-Whitney *U* test suggests that the difference between the mean values is significant (*P* < 0.05).

**Table 2:** Distribution of the teeth according to the level of repair seen and mean readings for level of repair

Group	Number	Score 0	Score 1	Score 2	Score 3			
		No repair	Partial repair	Functional repair	Anatomic repair	Mean	Standard deviation	*P* value
A	13	0	11	2	0	1.15	0.37	0.000*
B	15	0	0	6	9	2.6	0.5	

*P < 0.05 = significant

### Thickness of repair cementum

**Fig. 8:**
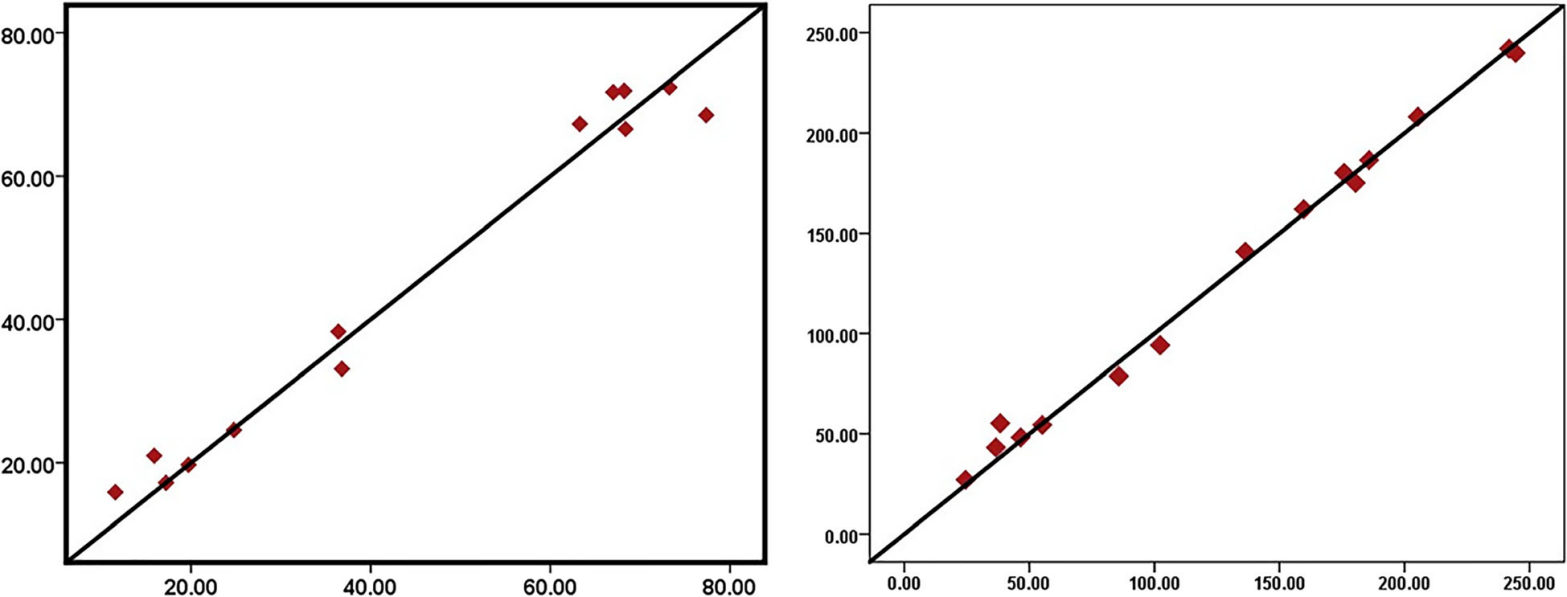
Agreement plot for the first and second operators. *X axis* readings by the first operator, *Y axis* readings by the second operator, *Left* plot for group A, *Right* plot for group B

Table 3 shows the correlation coefficients between the readings obtained by the two operators in both the groups. As seen here, there was a very high, statistically significant correlation (${r}_s$ close to +1) between the readings in both the groups. The agreement plots (Fig. 8) show the distribution of the readings. The mean thickness of repair cementum was 44.59 ± 25.81 μm in group A and 127.88 ± 77.19 μm as seen in Table 4. Mann-Whitney *U* test suggests that this difference between the thickness of cementum is significant (*P* < 0.05).

**Table 3:** Agreement between the two operators in both the groups

Group	Spearman’s rank correlation coefficient (*r*_*s*_)	*P* value
A	0.864	0.001*
B	0.986	0.000*

*P < 0.05 = significant

**Table 4:** Readings for thickness of repair cementum

Group	Number	Mean	Standard deviation	Median	Minimum reading	Maximum reading
A	13	44.59	25.81	36.79	11.57	77.34
B	15	127.88	77.19	136.31	24.46	244.34
*P* value		0.003*				

All readings in micrometer

*P < 0.05 = significant

## Discussion

Orthodontically induced inflammatory root resorption is an unavoidable side effect of orthodontic therapy. Minimizing this resorption by taking appropriate steps should be a moral duty for any orthodontist. That intermittent force is biologically less detrimental than continuous force is common knowledge [1, 2, 4, 5]. However, how intermittent this force should be is a question that still remains largely unanswered. This study was a small step towards understanding the same.

Intrusion is known to be the most harmful type of tooth movement where root resorption is concerned, with intrusive movement causing four times the resorption caused by extrusive movement [9]. Although the maxillary incisors have been shown to be the teeth most susceptible to resorption following intrusion [10], an in vivo study is possible only with premolars as they are the teeth most frequently extracted for orthodontic purpose [11, 12].

To standardize the morphology of the resorption craters for comparison in both the groups, the initial force levels, the duration, and the direction of the force were kept the same for both the groups. Following active intrusion, the teeth were retained with a passive spring instead of leaving them unbonded, avoiding resorption during extrusive relapse, as the objective was to study the repair of the craters induced by application of intrusive forces only.

There was a significant difference between the mean values for the level of repair in the two groups. Anatomic repair was seen more frequently in group B while partial repair was more frequent in group A. This indicates that healing of craters progresses rapidly with time. Previous studies have shown similar results. Be it tipping forces [3], or intrusive forces [6], level of repair was always shown to increase with time.

While a majority of the teeth in group B showed anatomic repair after a rest period of 6 weeks, there were still six teeth in this group that showed only functional repair. Based on these results, the hypothesis that full anatomic repair occurs with 6 weeks of force withdrawal was rejected. It can be concluded that 6 weeks is close to, but not enough for complete repair of orthodontic resorption. Six weeks, however, is enough for the exposed dentin to get fully covered with new cementum. At first glance at the previous studies, it might seem that full repair of orthodontic resorption occurs by 8 weeks. In the study by Cheng et al. [6], anatomic repair of craters was only seen in the teeth which were allowed to rest for a period of 8 weeks, irrespective of the levels of intrusive force applied to induce the resorption (25 or 225 g). However, the sample size in this study was too small to be conclusive. As stated earlier, Owman-Moll et al. [3] reported more frequent appearance of anatomic repair (12%) in the teeth retained for 8 weeks. The remaining 88% of the teeth in this group however did not show anatomic repair in spite of waiting for 8 weeks. Ahmed et al. [13] reported greater healing by 8 weeks even though the resorption was secondary to an acute trauma induced with mini-screw implants. They also reported only 73.1% repair even after a rest period of 12 weeks. The findings of the rat study by Gonzales et al. [14] suggest that minor resorption craters can be seen on the roots even after 16 weeks of retention. Ethical limitations, however, do not allow including such long rest periods in humans as it would mean delaying the orthodontic treatment in the subjects for longer periods. Thus, a definite time period at which full repair can be expected with confidence cannot be confirmed. A healing period of 6 to 8 weeks however does seem to fully repair most of the resorption craters or at least cover the entire surface of the exposed dentinal tubules at the bottom of all the resorption craters. Resorption and repair are thought to occur simultaneously in the presence of orthodontic load also [15]. The phenomenon that dominates this cyclic process decides if there will be irreversible damage to the roots. Intermittent rest periods of 6 to 8 weeks are recommended in between the orthodontic therapy to give repair a better chance over resorption.

Another interesting observation was that initial repair (group A) was predominantly acellular in nature while later repair proceeded with deposition of cellular cementum predominantly (group B). This finding is in agreement with previous studies [3, 6, 16]. This can be explained by the phenomena of non-functional retarded acellular repair and functional rapid cellular repair as discussed by Vardimon et al. [17]. Acellular cementum has been known to form more slowly [18]. To fill large voids as created by orthodontic forces, faster healing is required, which results in the cementocytes getting trapped within the lacunae before complete mineralization of the increments can take place.

For this study, it was hypothesized that with an increase in the time period allotted for the repair, the greater amount of cementum would be deposited within the resorption craters. If this hypothesis was true, then the thickness of repair cementum should have been greater in the teeth in group B as opposed to that in group A. There was a significant amount of difference between the mean values of the repair cementum thickness seen in both the groups, with the mean value clearly being more in group B (127.88 μm) as compared to that in group A (44.59 μm). This indicates that the amount of cementum deposited is indeed greater at 6 weeks. However, the standard deviation accompanying these values was very high (25.81 μm for group A, 77.19 μm for group B) with individual values ranging from 11.57 to 77.34 μm in group A and 24.46 and 244.34 μm in group B.

While the high and significant values of Spearman’s rank correlation coefficient in both the groups validate the reliability of the method used to measure the repair cementum thickness in this study, other factors may have played a role in causing this variability including (i) variations in root anatomy and surface morphology which can be seen between contralateral teeth also [19, 20], leading to different stress distribution in PDL. This can cause different levels and patterns of resorption on different areas of the root [21]; (ii) errors in the initial cut after decalcification, which divides the specimen into two for embedding into paraffin. Utmost care was taken to make this cut precisely through the marking made under the stereomicroscope. However, errors can occur during this procedure as it is a manual step and depends on the skill, accuracy, and the experience of the technician; and (iii) non-correlation of the area of the crater with its depth. The assumption was that the crater with the biggest surface area under a stereomicroscope would also be the deepest one, thus standardizing the depth of the craters. However, the surface area of the crater is a function of the area upon which the stresses are concentrated, which again depend on the morphology of the root surface as discussed earlier [21]. Multiple Howship’s lacunae may sometimes coalesce to form larger craters, as has been evident in other studies [22], thus increasing the surface area of the craters. The depth of the crater, on the other hand, is a function of the constancy of the force. When an orthodontic force is applied, the stresses generated at a particular point tend to reduce with time as the bone and cementum remodel around this point. The more constant the force, the deeper the resorption craters [5]. If these two factors are not correlated, then the depth of the craters was not truly standardized.

There were certain other limitations to this study. A cantilever spring such as the one used in this study would tend to exert extrusive and distal tipping forces on the molars. Future studies using a similar spring design could employ the use of anchorage preservation protocols, like a lingual holding arch for instance, to maximize the intrusion force on the premolars.

## Conclusions

While 6 weeks of rest period was not found to be adequate for full repair of the craters, it was surely enough for the exposed dentinal tubules to be fully covered with repair cementum. Repair of root resorption is a spontaneous, time-based phenomenon, with a longer rest period of 6 weeks showing better healing than a shorter rest period of 4 weeks. This repair begins at the centre of the resorption crater with deposition of acellular cementum and progresses further with deposition of cellular cementum extending to involve the entire surface of the crater. The thickness of the repair cementum also increases with time but is possibly only limited to the actual depth of the crater.
